# Fluoride Content and Its Potential Availability in Dentifrice Tablets Available on the Dutch market: A Laboratory Assessment

**DOI:** 10.1159/cre/abuag013

**Published:** 2026-09-29

**Authors:** Rob A.M. Exterkate, Bastiaan P. Krom, Catherine M.C. Volgenant

**Affiliations:** a Department of Preventive Dentistry, Academic Centre for Dentistry, Amsterdam, The Netherlands; b Department of Cariology, Academic Centre for Dentistry, Amsterdam, The Netherlands

**Keywords:** Dentifrices, Dentifrice tablets, Fluorides, Potentially available fluoride

## Abstract

**Introduction:**

Dentifrice tablets are increasingly presented as a more sustainable alternative to regular toothpastes, yet evidence regarding their efficacy is still limited. This study aimed to evaluate the quality of dentifrice tablets available on the Dutch market with respect to their total fluoride content and their potentially available fluoride.

**Methods:**

Twenty types of dentifrice tablets were analyzed using two different techniques: an ion-selective electrode and gas-liquid chromatography. Prior to analysis, two criteria were defined: (1) total content of fluoride should be >80% of the content that the manufacturer claimed and (2) the potentially available fluoride content should be ≥80% of the measured total fluoride content.

**Results:**

Nine of the twenty tablets met both criteria. Moreover, most tablets had a lower weight than the amount of toothpaste recommended by the Dutch Ivory Cross. This variation in quality, combined with the relatively lower mass of dentifrice tablets raises concerns regarding the reliability of dentifrice tablets for caries prevention.

**Conclusion:**

Although these tablets were purchased on the Dutch market, the results of this study suggest that dentifrice tablets in general may lack sufficient quality control.

## Introduction

Caries is still worldwide one of the most common diseases in the oral cavity. Despite continuous efforts to reduce caries, only 21% of Dutch 23-year olds are caries-free [1]. Fluoride is still the cornerstone of caries prevention. Brushing twice daily with a fluoride-containing dentifrice is a key component of prevention strategies in the Netherlands [2]. There are many types and brands of dentifrices available, and the fluoride source varies considerably. Sodium fluoride, amine fluoride, stannous fluoride, and monofluorophosphate are the most common sources of fluoride. Besides fluoride, other components are added to modern dentifrices for among other plaque control or tooth whitening. However, fluoride remains the key component in dentifrices, with an age-dependent advised concentration. In the Netherlands, dentifrices with 500–750 ppm fluoride are recommended for children up to 4 years of age and 1,000–1,500 ppm fluoride is advised for people aged 4 years and older [2].

Nowadays, more and more dentifrice tablets are introduced on the market. These tablets are claimed to be a more sustainable alternative to traditional dentifrices. While packaging waste may be reduced and water-free formulations ease transport, a comparative life-cycle assessment found a slightly higher overall 6-month impact for tablets than paste, largely from the use phase and specific tablet excipients; recycling of glass jars offers partial mitigation [3]. Dentifrice tablets come in portioned quantities which can be used completely, whereas regular dentifrice tubes are difficult to empty completely and, although they are increasingly recycled, tubes tend to end up in nonrecyclable waste.

At the same time, dentifrice tablets present a potential problem. Manufacturers of traditional dentifrices invest a lot of time and knowledge in maintaining all the components in a stable and effective condition and in gel/liquid form. But dentifrice tablets merely contain all their components in dry form, which are pressed into a tablet. The tablet needs to be chewed after which the individual components need to dissolve within the short time frame of a brushing session, typically 1–2 min.

For caries prevention, it is important that the fluoride in the dentifrice is sufficiently available in the oral cavity. Fluoride can be present in an ionic or ionizable from, which is called potentially available [4] or can be bound in the dentifrice which makes the fluoride unavailable for caries prevention. Current general standards for dentifrices (e.g., NEN-EN-ISO 11609) do not require a minimum level of potentially available fluoride, while national recommendations differ in their advised fluoride concentrations. In general, regular toothpastes meet these requirements, but a number of publications have shown that potentially available fluoride can be low/too low in regular toothpastes [5–8].

Although dentifrice tablets currently hold a small percentage of the overall market, their presence is increasing. Initially, these tablets were mainly sold through internet-based ecological companies, but nowadays, they can be purchased in supermarkets and drugstores. The number of brands and points of sale is growing rapidly. Therefore, it is important to establish the quality of dentifrice tablets with respect to fluoride to adequately advise patients regarding caries prevention. The present study therefore measured the total fluoride content and potentially available fluoride content of twenty of dentifrice tablets available on the Dutch market.

## Methods

### Study Design and Product Selection

Twenty different types/brands of dentifrice tablets were purchased in physical and internet-based shops between January and March 2025. For each product, the total fluoride content and the potentially available fluoride content were determined using two generally used technologies: the ion-selective electrode (ISE) and gas-liquid chromatography (GLC) [9–11]. Prior to analysis, two criteria were defined: 1) total fluoride content should be >80% of the content claimed by the manufacturer and 2) the potentially available fluoride content should be ≥80% of the measured total fluoride. A dentifrice tablet only qualified as adequate if both criteria were met.

### The Preparation of Tablet Slurries

The advised use of each product (e.g., one tablet per brushing) and the average tablet mass were recorded. For subsequent analyses, multiple tablets per product were used to reach a total weight of approximately 1.0 g. The actual weight was measured with a 1 mg accuracy. The tablets were pulverized and suspended in 10 mL ultrapure water (Milli-Q, Merck-Millipore, The Netherlands). This mixture was mixed by hand for 1 min and subsequently mixed on a magnetic stirrer for 4 min to obtain a homogenous slurry. For the determination of potentially available fluoride, 1 mL of slurry was transferred to 4, 1.5-mL reaction tubes (Sarstedt, Nümbrecht, Germany) in quadruplicate and centrifuged for 2 min (21,330 *g*). The supernatant of three of the tubes was combined and used for the ISE, the fourth tube was used for the GLC. For the determination of the total fluoride content, the unprocessed slurry was used. For each type of tablet, six independent slurries were prepared; three for the determination of potentially available fluoride and three for the determination of total fluoride.

### Measurement with the ISE

#### Potentially Available Fluoride

One milliliter of supernatant was diluted with 2 mL of ultrapure water. From this mixture, 2 mL was mixed with 2 mL TISAB II (4 g Titriplex IV buffer [Merck, Darmstadt, Germany], 57 mL glacial acetic acid, 59 g NaCl per liter, pH 5.0) and measured directly with the ISE (perfectION comb F, Mettler-Toledo, The Netherlands). Some tablets contained monofluorophosphate as a fluoride source. The diluted supernatants of these tablets required an additional enzymatic step to free the fluoride. The diluted supernatant (0.5 mL) was incubated with 0.5 mL acid phosphatase (Sigma-Aldrich, P3627; 8 units/mL) in a buffer (7.3 g/L sodium acetate, 6.6 g/L glacial acetic acid, pH 4.8) [10]. After 24 h, 1 mL of TISAB II was added and the mixture was measured with the ISE.

#### Total Fluoride

In order to measure the total fluoride content with the ISE, all the fluoride should be in ionic form. The method of Taves [12] was used. In a standard disposable Petri dish, a small cup with 100 μL 1.65 mol/L NaOH was placed. The 400 μL tablet slurry and 7 mL 3.4 mol/L perchloric acid were added to the Petri dish. The lid was sealed with petroleum jelly (Vaseline) and 1 mL of 6 mol/L HCl saturated with hexamethyldisiloxane was added through a small hole which was immediately sealed off with petroleum jelly. The concept is that fluoride reacts with HMDS in an acid environment and forms TMFS (trimethylfluorosilane) which vaporizes and will fall apart in the alkaline environment in the cup with NaOH. After 24 h, the small cup with NaOH was placed in a stove at 37°C and left to dry for 24 h. After addition of TISAB II, the solution could be measured using the ISE. A range of fluoride standards underwent the same procedure.

### Measurement with GLC

Both slurries, for total and potentially available fluoride, were analyzed as described below. A GLC supplied with an auto-sampler is used for the analysis (Gas chromatograph, model GC-2010Plus, autoinjector AOC-20i, autosampler AOC-20 s, Shimadzu, Japan) The autosampler is cooled at 10°C using a circulation cooler. (Circulation cooler Neslab RTE-101, Thermo-Fisher, The Netherlands). The fluoride analysis with the GLC is based on the work of Fresen et al. [13] based on the principle that fluoride reacts with trimethylchlorosilane (TMCS) in an acidic environment to form trimethylfluorosilane (TMFS). Both TMCS and TMFS are insoluble in water. A toluene reagent was prepared by mixing 100 μL TMCS, 9 mL toluene, and 900 μL iso-pentane (0.1% iso-pentane in toluene). Iso-pentane was used as an internal standard to correct for small differences in injection volume of the GLC.

The toluene reagent (400 μL) was incubated with a mixture of 400 μL solution (tablet slurry, supernatant, or standard solution) and 1 mL 1 mol/L HCl in a gastight container. The container was placed overnight in an overhead shaker at 4°C. The next day, the container was centrifuged at low speed (1,400 rpm) to separate the toluene and water phase. The autoinjector of the GLC injects 1 μL of the toluene phase into the GLC in duplicate. The area of the TMFS and iso-pentane peaks is determined by the software (LabSolutions, version 5.97, Shimadzu, Japan).

### Calibration and Quality Control

Both methods use a calibration curve with known concentrations of fluoride. The preparation of the dentifrice tablet samples was the same for both technologies, and only different in details necessary for the technology used. Primary outcomes were: (i) potentially available fluoride (µg/g) and (ii) total fluoride (µg/g), using ISE and GLC. To determine the quality of the dentifrice tablets, potentially available fluoride was also expressed as % of measured total fluoride and measured total fluoride as % of the manufacturer’s claim. Products were considered to meet predefined quality criteria if both preset criteria were achieved.

### Statistics

The data obtained from the ISE and GLC samples were tested for differences between the methods using the related-samples Wilcoxon signed rank test (SPSS 29.0.1.0, IBM).

## Results

For all tested tablets dentifrice tablets data were obtained in triplicate for both analytical techniques. In total, 60 sets of data for the potentially available fluoride and the total fluoride were available. The data obtained with both techniques showed no statistical difference using the Wilcoxon related-samples test.

For all dentifrice tablets, the potentially available and total fluoride concentration were determined using both ISE and GLC. In Table 1, the average and standard deviation of the measured fluoride concentrations are presented. In the column “% potentially available versus total F,” the potentially available fluoride is expressed as a percentage of the measured total fluoride content. In the column “% total F versus claimed” the measured total fluoride is expressed as percentage of the content claimed by the manufacturer. For both the ISE and the GLC, the percentages were calculated and the averaged outcome of the two technologies is presented in Table 1.

**Table 1.** Average weight (g) and fluoride concentrations (µg/g; mean and standard deviation; *n* = 3) of dentifrice tablets available on the Dutch market

​		Source of fluoride	Weight of tablet (g)	Claimed by supplier	Ion selective electrode	Total F (µg/g)	Gas chromatograph	Total F (µg/g)	% total F versus claimed*	% potentially available versus total F*
					potentially available F (µg/g)		potentially available F (µg/g)			
Adequate										
Choose	Choose TeethCare BV	MFP	0.40 (0.01)	1,450	1,556 (64)	1,551 (228)	1,499 (34)	1,301 (25)	98	108
Denttabs mint	Denttabs GmbH	NaF	0.31 (0.00)	1,450	1,163 (241)	1,326 (87)	1,164 (220)	1,286 (54)	90	89
Denttabs strawberry (child)	Denttabs GmbH	NaF	0.31 (0.00)	500	549 (5)	479 (18)	498 (51)	439 (2)	92	114
Humble brush	The Humble Co.	MFP	0.44 (0.08)	1,450	1,485 (22)	1,498 (405)	1,350 (138)	1,255 (47)	95	103
I + M	Denttabs GmbH	NaF	0.32 (0.00)	1,450	1,496 (19)	1,280 (421)	1,619 (18)	1,232 (21)	87	124
Smyle Kids (child)	Smyle	MFP	0.30 (0.05)	1,000	1,038 (110)	1,036 (183)	1,055 (103)	1,015 (61)	103	102
Smyle original	Smyle	MFP	0.35 (0.05)	1,450	1,402 (73)	1,321 (282)	1,385 (83)	1,231 (28)	88	109
Smyle sensitive	Smyle	MFP	0.32 (0.06)	1,450	1,417 (147)	1,366 (103)	1,564 (103)	1,316 (26)	92	111
Smyle whitening	Smyle	MFP	0.33 (0.05)	1,450	1,238 (48)	1,249 (297)	1,264 (89)	1,216 (31)	85	102
Too low total F (<80%)										
Happy soaps	HappySoaps.com	NaF	0.65 (0.00)	1,450	954 (212)	1,034 (325)	1,037 (178)	1,193 (37)	**77**	90
Smyle salty fresh	Smyle	MFP	0.39 (0.08)	1,450	1,319 (239)	902 (258)	1,249 (130)	1,050 (20)	**67**	133
T-brush charcoal	Tuzz	NaF	0.45 (0.04)	1,400	446 (29)	525 (239)	630 (90)	539 (65)	**38**	101
T-brush mint	Tuzz	NaF	0.45 (0.00)	1,400	586 (44)	489 (63)	773 (233)	540 (3)	**37**	131
Lazea V34	Lazea Cosmetics	NaF	0.69 (0.01)	1,500	13 (0)	15 (5)	42 (20)	69 (8)	**3**	75
Dental Care (Albert Heijn)	Albert Heijn BV	NaF	0.61 (0.01)	1,450	458 (94)	1,179 (356)	445 (79)	1,053 (120)	**77**	**41**
Too low potentially available F (<80%)										
Etos	Etos BV	NaF	0.60 (0.01)	1,450	299 (72)	1,276 (330)	318 (17)	1,054 (93)	80	**27**
Happy tabs	Happy-Tabs.com	NaF	0.60 (0.01)	1,200	326 (24)	969 (244)	333 (33)	1,054 (101)	84	**33**
Lamazuna ultra fresh	Lamazuna SAS	NaF	0.25 (0.00)	1,350	501 (30)	1,170 (111)	480 (17)	1,233 (103)	89	**41**
Lamazuna wild mint	Lamazuna SAS	NaF	0.26 (0.03)	1,450	572 (52)	1,452 (451)	549 (75)	1,201 (46)	91	**43**
Oceonics	Oceonics.nl	NaF	0.33 (0.04)	1,450	826 (108)	1,403 (283)	995 (220)	1,296 (23)	93	**68**

Fluoride was measured by ion-selective electrode (ISE) and gas-liquid chromatography (GLC). Potentially available fluoride is expressed as “% potentially available versus total F.” The measured total fluoride is expressed as percentage of the fluoride concentration claimed by the manufacturer “% total versus claimed.” The percentages were calculated using the outcome of both techniques and the average percentage of the two techniques are presented in the table. *Calculated from the average result of the two methods In bold are the outcomes that are below the predefined criteria regarding F (total fluoride ≥ 80% of the manufacturer's claim and potentially available fluoride ≥ 80% of the measured total fluoride).

Nine out of twenty tested tablets met both predefined criteria regarding total fluoride ≥80% of the manufacturer’s claim and potentially available fluoride ≥80% of the measured total fluoride. Three tablets showed substantially lower total fluoride than claimed (Lazea V34, T-brush Charcoal and T-brush mint) and two tablets scored borderline (HappySoaps and Smyle salty fresh). Nine tablets showed insufficient potentially available fluoride; in seven of these, the percentage of potentially available versus total fluoride was low. In the two T-brush products, the absolute potentially available fluoride concentration was below the threshold for an adult dentifrice product.

Average tablet mass and within-brand variability are also presented in Table 1. Tablet mass differed between the brands and showed small standard deviations within brands. Fifteen of the twenty products had an average mass below the in-the-Netherlands-recommended 0.6–1.2 g dentifrice amount per brushing session, indicating that the total intraoral fluoride dose delivered per brushing is often lower than advised.

Two products were marketed for children. One (Smyle Kids) had a fluoride content too high for children aged <5 years according to the local Dutch advice. But this product has a concentration in line with the advice in other countries and the advice from the EAPD [14]. Denttabs Strawberry had a sufficient fluoride level, but a strong strawberry flavor, which may encourage unintended consumption of the tablets.

## Discussion

This study shows that the quality of dentifrice tablets with respect to fluoride content varies considerably. Less than half of the tablets tested contained sufficient potentially available fluoride as well as a total fluoride content in agreement with the claimed amount by the manufacturer. It is also evident that the weight of most tablets is low, as compared to the advised amount of dentifrice per toothbrushing session. In the Netherlands, it is advised to use 1–2 cm of toothpaste [2], representing appr. 0.6–1.2 g of toothpaste. Fifteen of the twenty tablets had a lower weight than this advised amount indicating that by using tablets, the total amount of fluoride becoming intraorally available is lower than advised. There are only a few reports that address the issue of amount of dentifrice with respect to caries prevention [15–18]. However, it seems self-evident that the amount of dentifrice used does play a role in caries prevention. There might be a difference in the way that dentifrice tablets actually get diluted by saliva in the mouth. Chewing a tablet may stimulate the salivary flow, which could influence the dilution and distribution of available fluoride. At present, no studies have compared intraoral fluoride concentrations after using dentifrice tablets and regular toothpaste.

Both observations, insufficient potentially available fluoride and low average weight, raise questions with respect to the expected caries-preventive effect of dentifrice tablets and emphasize that quality control of these innovative dentifrice tablets should be improved. At the same time, current legislation lacks proper control of the required level of potentially available fluoride in preventive products.

In the past, studies already showed that toothpastes in non-European markets sometimes lack the expected quality [3–6], and the present findings suggest that a notable proportion of dentifrice tablets available in the Netherlands may also fall short of expected levels. Further research is needed to determine what the quality of dentifrice tablets available in other countries is. In addition, the international scientific community should work on the adaptation of current guidelines to at least address the issue of fluoride availability in both traditional toothpaste and newly developed dentifrice tablets. Levine [19] advocated that the most reliable indicator of the effective fluoride content for a toothpaste formulation is the ionic fluoride concentration (F−) as parts per million available immediately on brushing and that this concentration should be on the product label. But until now, his plea was not followed by regulatory measures.

With respect to the technologies used to measure the fluoride levels, both techniques showed a high agreement. Fluoride measurements with the ISE have the advantage of a low investment in equipment. However, it may take more time and effort to perform the analysis. Electrode measurements may take up to 10 min to get a stable signal and cannot be automated easily by using an autosampler. And especially the analysis of total fluoride, using the method described by Taves [11], requires more time and is more prone to errors due to the more complicated handling of the sample. The GLC has the advantage that the preparation of samples does not take a lot of time and the analysis can be automated. Of course, this requires a higher investment in the equipment. But once the samples are ready, the analysis can be performed without monitoring. A workgroup of the ORCA (Organization for Caries Research) has produced an extensive overview of methods for the determination of fluoride with pros and cons of the methods [4]. The method that is chosen depends on the required investment or even the researcher’s preference, but both the ISE and GLC are considered to be adequate methods for the analysis of fluoride content. The fact that sometimes potentially available concentrations exceed the measured total fluoride content reflects sample-preparation variability. These high values were all interpreted as indicating complete or near-complete availability rather than true availability above total fluoride.

Currently, a fluoride level of 1,000–1,500 ppm is advised in dentifrices for adults in The Netherlands. From the current dataset, it can be concluded that manufacturers aim to achieve fluoride levels in that range based on their claimed fluoride content, but a substantial number of dentifrice tablets do not meet those levels of fluoride as potentially available fluoride. In those cases, tablets show levels of potentially available fluoride well below the 1,000 ppm threshold, which makes them not suitable for adults. The question remains why the fluoride which is in the product is not released as ionic fluoride. A part of the answer could be that tablets contain compounds like CaCO_3_ that bind the fluoride and makes the fluoride partly unavailable.

In the current dataset, there are two tablets aimed at children of which Smyle Kids has a too high concentration for children aged <5 years according to the Dutch advice. But the concentration is in line with the advice of the EAPD [14]. The Denttabs Strawberry does have a lower fluoride content (500 μg/g) which is in line with the Dutch advice but lower than the amount advised by the EAPD. Both tablets weigh (0.30 g) some 20% more than the advised weight by the EAPD (0.25 g for children up to 6 years). When children are young, one should wonder if they are able to chew and spread the tablet in the oral cavity properly before they brush. And especially when the tablet has an attractive taste, it might be considered as candy by young children resulting in excessive consumption of the product. Taken together, extreme care should be taken when marketing these tablets to children.

In the current study, only fluoride content and release were measured. Other potentially beneficial compounds were not tested for their release and availability, so their contribution to caries prevention remains unclear. Dentifrice tablets are relatively new on the market and research on their efficacy is limited to a few in vitro studies or short-term clinical studies [20, 21]. One could argue that when dentifrice tablets release their compounds properly and these compounds are available, their efficacy should be comparable to the same compounds in a regular toothpaste. However, at present, it seems that caries prevention would benefit from improved quality control of the production of dentifrice tablets, both by manufacturers and the scientific community.

## Conclusion

The quality of dentifrice tablets available on the Dutch market varies with respect to their fluoride content and levels of potentially available fluoride. Less than half of the tested tablets had sufficient quality. In addition, most dentifrice tablets had a relatively low weight compared to the advised amount of toothpaste. Both observations raise questions about the expected caries-preventive efficacy of these new products in everyday use.

Unless quality control is improved, a general recommendation for tablets as a suitable alternative for regular toothpaste cannot be given. It is strongly advised for manufacturers to routinely test their tablets, before launching them on the consumer market.

## Acknowledgments

The authors thank ACTA dental students Anne Landwer Johan and Rosemarijn van de Woestijne for their contribution to this study.

## Statement of Ethics

An ethics statement was not required for this study type since no human or animal subjects or materials were used.

## Conflict of Interest Statement

Authors have no conflict of interest. The dentifrice tablets were selected based on their availability on the Dutch market and were bought in web shops or regular supermarkets/drugstores. None of the producers were involved in any way in the described study.

## Funding Sources

This research received no external funding.

## Author Contributions

R.A.M.E. designed the study and performed the analyses. All authors contributed to the interpretation of the findings. R.E. drafted the manuscript, and B.P.K. and C.M.C.V. critically revised the text. All authors approved the final version of the manuscript.

## Data Availability

The data that support the findings of this study are not publicly available due to privacy reasons but are available from the corresponding author upon request.
